# MicroRNA-193a-3p as a Valuable Biomarker for Discriminating between Colorectal Cancer and Colorectal Adenoma Patients

**DOI:** 10.3390/ijms25158156

**Published:** 2024-07-26

**Authors:** Marija Fabijanec, Andrea Hulina-Tomašković, Mario Štefanović, Donatella Verbanac, Ivana Ćelap, Anita Somborac-Bačura, Marija Grdić Rajković, Alma Demirović, Snježana Ramić, Božo Krušlin, Lada Rumora, Andrea Čeri, Martha Koržinek, József Petrik, Neven Ljubičić, Neven Baršić, Karmela Barišić

**Affiliations:** 1 Center for Applied Medical Biochemistry, Faculty of Pharmacy and Biochemistry, University of Zagreb, 10000 Zagreb, Croatia; marija.fabijanec@pharma.unizg.hr; 2 Department of Medical Biochemistry and Hematology, Faculty of Pharmacy and Biochemistry, University of Zagreb, 10000 Zagreb, Croatia; mstefan6@gmail.com (M.Š.); donatella.verbanac@pharma.unizg.hr (D.V.); ivana.celap@gmail.com (I.Ć.); anita.somborac@pharma.unizg.hr (A.S.-B.); marija.grdic@pharma.unizg.hr (M.G.R.); lada.rumora@pharma.unizg.hr (L.R.); andrea.ceri@pharma.unizg.hr (A.Č.); martha.korzinek@pharma.unizg.hr (M.K.); jozsef.petrik@pharma.unizg.hr (J.P.); karmela.barisic@pharma.unizg.hr (K.B.); 3 Department of Clinical Chemistry, Sestre Milosrdnice University Hospital Center, 10000 Zagreb, Croatia; 4 Department of Pathology and Cytology “Ljudevit Jurak”, Sestre Milosrdnice University Hospital Center, 10000 Zagreb, Croatia; demirovic.alma80@gmail.com (A.D.); bozo.kruslin@gmail.com (B.K.); 5 Department of Oncological Pathology, University Hospital for Tumors, Sestre Milosrdnice University Hospital Centre, 10000 Zagreb, Croatia; snjezana.ramic@gmail.com; 6 Department of Internal Medicine, Division of Gastroenterology and Hepatology, Sestre Milosrdnice University Hospital Center, 10000 Zagreb, Croatia; neven.ljubicic@kbcsm.hr (N.L.);

**Keywords:** colorectal cancer, colorectal adenoma, colonoscopy, liquid biopsy, CEA, CA 19-9, microRNA, miR-193a

## Abstract

Specific markers for colorectal cancer (CRC), preceded by colorectal adenoma (pre-CRC), are lacking. This study aimed to investigate whether microRNAs (miR-19a-3p, miR-92a-3p, miR-193a-3p, and miR-210-3p) from tissues and exosomes are potential CRC biomarkers and compare them to existing biomarkers, namely carcinoembryonic antigen (CEA) and carbohydrate antigen (CA) 19-9. MiRNA was isolated in the samples of 52 CRC and 76 pre-CRC patients. Expression levels were analyzed by RT-qPCR. When comparing pre-CRC and CRC tissue expression levels, only miR-193a-3p showed statistically significant result (*p* < 0.0001). When comparing the tissues and exosomes of CRC samples, a statistically significant difference was found for miR-193a-3p (*p* < 0.0001), miR-19a-3p (*p* < 0.0001), miR-92a-3p (*p* = 0.0212), and miR-210-3p (*p* < 0.0001). A receiver-operating characteristic (ROC) curve and area under the ROC curve (AUC) were used to evaluate the diagnostic value of CEA, CA 19-9, and miRNAs. CEA and CA 19-9 had good diagnostic values (AUCs of 0.798 and 0.668). The diagnostic value only of miR-193a-3p was highlighted (AUC = 0.725). The final logistic regression model, in which we put a combination of CEA concentration and the miR-193a-3p expression level in tissues, showed that using these two markers can distinguish CRC and pre-CRC in 71.3% of cases (AUC = 0.823). MiR-193a-3p from tissues could be a potential CRC biomarker.

## 1. Introduction

### 1.1. Incidence and Mortality of CRC

Colorectal cancer (CRC) is a growing health concern worldwide, with increasing incidence and mortality rates. It is currently the third leading cause of death among malignant diseases. Raising awareness and supporting the prediction, prevention, and research efforts are crucial to fight this devastating disease [1]. In Croatia, 6 people die every day due to CRC, with 10 new cases being diagnosed daily [2].

### 1.2. Searching for New Markers

The development of CRC is a complex process; it involves the transition from benign adenoma to malignant adenocarcinoma and eventually the spread to distant areas through metastasis. Several genetic and epigenetic factors contribute to the development of CRC by affecting cellular and signaling pathways [3]. Since CRC progresses from a benign to a malignant tumor over time, early detection is critical to successful treatment. Patients diagnosed in stages I and II have a remarkable 90% survival rate over five years. However, the prognosis drops to 71% in patients with lymph node metastasis and an alarming 13% in those with distant metastases. Therefore, it is imperative to prioritize regular screenings and early detection to increase chances of survival [4].

Nevertheless, approximately 60% of cases are not detected until they have progressed to advanced stages (III and IV) of the disease, which is linked to a negative outcome and an increase in mortality [5]. Different ways to detect CRC include non-invasive stool analysis to check for hidden blood and invasive endoscopic examination like colonoscopy.

Over the past few decades, researchers have studied many molecules as potential markers for CRC. Some of the established protein markers that can be detected in peripheral blood include carcinoembryonic antigen (CEA), embryo-specific glycoprotein, and carbohydrate antigen 19-9 (CA 19-9) [6,7,8]. After the removal of cancerous cells, monitoring the levels of CEA can be helpful in detecting any signs of metastatic disease [9,10,11].

New markers with better diagnostic potential are being extensively researched to detect the disease at an early stage. This will facilitate the search procedure using the least invasive form of taking a biological sample, as the current markers have limited diagnostic potential.

The latest research has yielded exciting results in the hunt for more effective markers. MicroRNAs are short (19–25 nucleotides) non-coding regulatory transcripts that have emerged as promising candidates that could lead to groundbreaking discoveries [12]. MicroRNAs are molecules that control gene expression at the post-transcriptional co-translational level [13]. It has been shown that they are involved in numerous processes leading to tumor formation and their diagnostic potential seems promising in the case of CRC [14,15,16,17].

### 1.3. The Concept of Liquid Biopsy

Liquid biopsy is a diagnostic procedure that involves analyzing a biological sample obtained from a liquid, such as peripheral blood. It acts as a “window” through which we can observe what is happening in the body [18]. This non-invasive method is used to detect the existence of malignant tumors like CRC and determine their molecular properties, including gene, RNA, and protein profiles. By analyzing these properties, relevant data can be obtained, which help in diagnosing the disease and deciding on the best therapeutic protocols [19].

### 1.4. Applying the Challenges of Modern Oncology Diagnostics

We conducted research on potential markers for CRC among four microRNAs: miR-19a, miR-92a, miR-193a, and miR-210. Our study involved analyzing their expression in adenoma and carcinoma tissues of recruited patients, obtained through colonoscopy, and in derivatives of whole blood, obtained through liquid biopsy, i.e., in exosomes isolated from plasma. To achieve the primary goals, we aimed to (i) determine and compare microRNA expressions in tissues and circulation and (ii) compare their diagnostic potential with already established tumor biomarkers and histological features of the tissue. The following is a brief description of the specific characteristics and features of these molecules of interest.

MiR-19a is a component of the miR-17–92 cluster, which is a primary transcript that yields six mature miRNAs: miR-17, miR-18a, miR-19a, miR-20a, miR-19b, and miR-92a. It may serve as a possible progression and prognostic biomarker for gastrointestinal malignancy [20].

MiR-92a is a key oncogenic component of the miR-17–92 cluster in colon cancer that directly targets the anti-apoptotic molecule BCL-2-interacting mediator of cell death in colon cancer tissue. Antagomir induces apoptosis in colon cancer-derived cell lines [21].

MiR-193a is located on chromosome 17 and is cleaved into mature miR-193a-3p and miR-193a-5p during its processing. It plays a role in various types of cancer, mostly as a tumor suppressor. In CRC, it inhibits the proliferation of cancer cells and induces apoptosis. Its overexpression has been shown to be associated with disease progression, but it has also been downregulated in colorectal cancer tissue and linked to prognosis [22,23].

MiR-210 is often upregulated in carcinogenesis and can encourage the migration and invasion of cancer cells. It can be induced by hypoxia and mediate hypoxia-induced metastasis [24]. It has been described as a potential non-invasive marker for diagnosis and prognosis of CRC [25].

## 2. Results

### 2.1. Patients’ Characteristics

This study included 52 CRC patients and 76 pre-CRC patients, which we used as a control group. We examined the age distribution of pre-CRC and CRC patients and, as expected, the patients were mostly older adults (67 years (32–84) for pre-CRC and 72 years (37–90) for CRC). The concentrations of tumor marker CEA were measured in 67 pre-CRC and 49 CRC serum samples, whereas CA 19-9 concentrations were measured in 67 pre-CRC and 48 CRC serum samples (Table 1).

All patients underwent pathohistological characterization. Pathohistological data for CRC patients (Table 2) were included in the statistical analysis to examine whether advanced stages of the cancer are associated with increased or decreased concentrations of CEA and CA 19-9 or expressions of the four chosen miRNAs: miR-19a-3p, miR-92a-3p, miR-193a-3p, and miR-210-3p. Unfortunately, we did not find statistically significant results when comparing standard tumor marker concentrations or miRNA expression levels and pathohistological parameters.

### 2.2. The Potential of CEA and CA 19-9 Concentrations in Discriminating against Pre-CRC and CRC Patients

**Figure 1:**
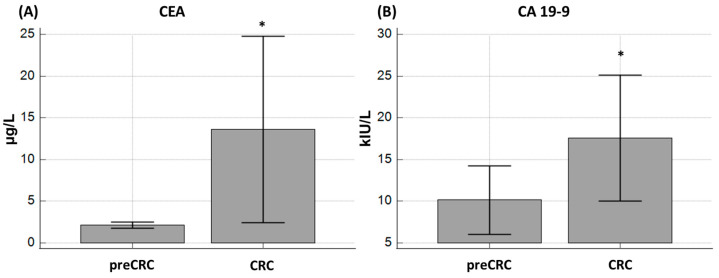
Serum concentrations of the tumor markers (**A**) CEA and (**B**) CA 19-9 between pre-CRC and CRC patients. A statistically significant difference was found both for CEA (*p* < 0.0001) and CA 19-9 (*p* = 0.0022). * Represents *p* < 0.05.

We found a statistically significant difference for CEA (*p* < 0.0001) and CA 19-9 (*p* = 0.0022) in the serum of pre-CRC and CRC patients. Concentrations of both tumor markers were higher in the CRC group (Figure 1).

**Figure 2:**
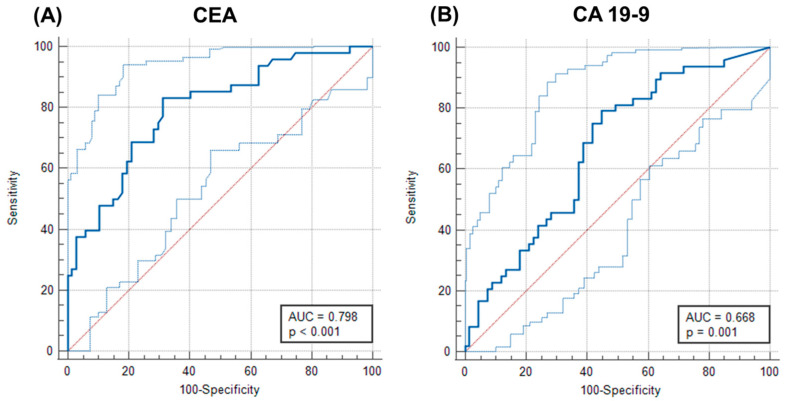
The ROC curves for (**A**) CEA and (**B**) CA 19-9 for CRC and pre-CRC patients. It was found that CEA and CA 19-9 are valuable markers for discriminating CRC patients from pre-CRC patients with AUCs of 0.798 (95% CI = 0.713–0.867, *p* < 0.0001) and 0.668 (95% CI = 0.574–0.753, *p* = 0.0009). The dark blue curve represents the receiver-operating characteristic (ROC) curve. The light blue curve represents a 95% ROC confidence interval. The red line represents a diagonal.

The diagnostic utility of CEA and CA 19-9 was evaluated by the ROC curve and area under the ROC curve (AUC) analysis for CRC and pre-CRC patients. We found that these two markers used in clinical practice for the prognosis and prediction of CRC are valuable markers for discriminating CRC patients from pre-CRC patients with AUCs of 0.798 (95% CI = 0.713–0.867, *p* < 0.0001) and 0.668 (95% CI = 0.574–0.753, *p* = 0.0009). CEA had higher specificity and sensitivity than CA 19-9 (Figure 2).

### 2.3. The Potential of miRNA Expression Levels in Discriminating against Pre-CRC and CRC Patients

**Figure 3:**
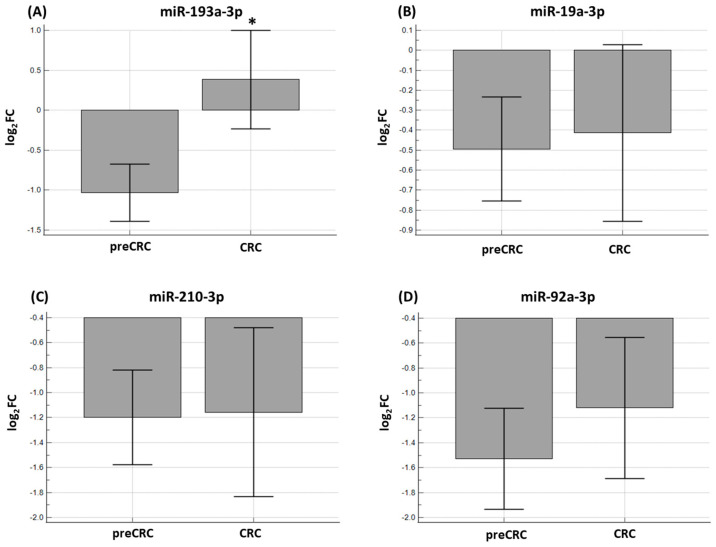
The expression levels between 76 pre-CRC and 52 CRC FFPE tissue samples for (**A**) miR-193a-3p, (**B**) miR-19a-3p, (**C**) miR-210-3p, and (**D**) miR-92a-3p. Only miR-193a-3p (*p* < 0.0001) showed a statistically significant difference and there was no statistically significant difference between the two groups for miR-19a-3p (*p* = 0.4220), miR-210-3p (*p* = 0.3656), and miR-92a-3p (*p* = 0.2413). log_2_FC—log_2_fold change. * Represents *p* < 0.05.

The expression levels of miR-19a-3p, miR-92a-3p, miR-193a-3p, and miR-210-3p were first compared in formalin-fixed paraffin-embedded (FFPE) tissues between pre-CRC and CRC patients. Only miR-193a-3p (*p* < 0.0001) showed statistically different expressions and there was no statistically significant difference between the two groups for miR-19a-3p (*p* = 0.4220), miR-92a-3p (*p* = 0.2413), and miR-210-3p (*p* = 0.3656) (Figure 3).

**Figure 4:**
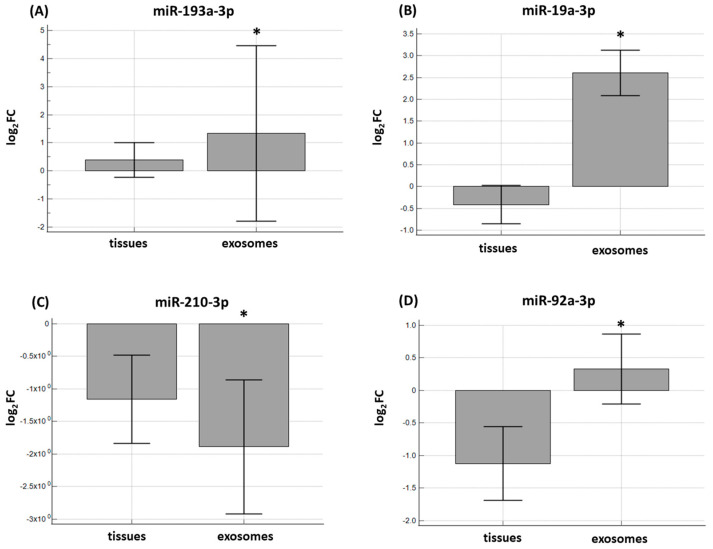
The expression levels of (**A**) miR-193a-3p, (**B**) miR-19a-3p, (**C**) miR-210-3p, and (**D**) miR-92a-3p between FFPE tissue samples and paired exosomes of 52 CRC patients. A statistically significant difference was found for miR-193a-3p (*p* < 0.0001), miR-19a-3p (*p* < 0.0001), miR-210-3p (*p* < 0.0001), and miR-92a-3p (*p* = 0.0212) as well. Log_2_FC—log_2_fold change. * Represents *p* < 0.05.

In addition, we investigated whether there was an association between the expression levels of miRNAs in the FFPE tissue samples and in paired exosomes isolated from the plasma of 52 CRC patients. MiR-92a-3p and miR-19a-3p were absent in six exosome samples. MiR-193a-3p was detected only in 10 and miR-210-3p in 18 exosome samples. A statistically significant difference was found for miR-193a-3p (*p* < 0.0001), miR-19a-3p (*p* < 0.0001), miR-92a-3p (*p* = 0.0212), and miR-210-3p (*p* < 0.0001) as well (Figure 4).

**Figure 5:**
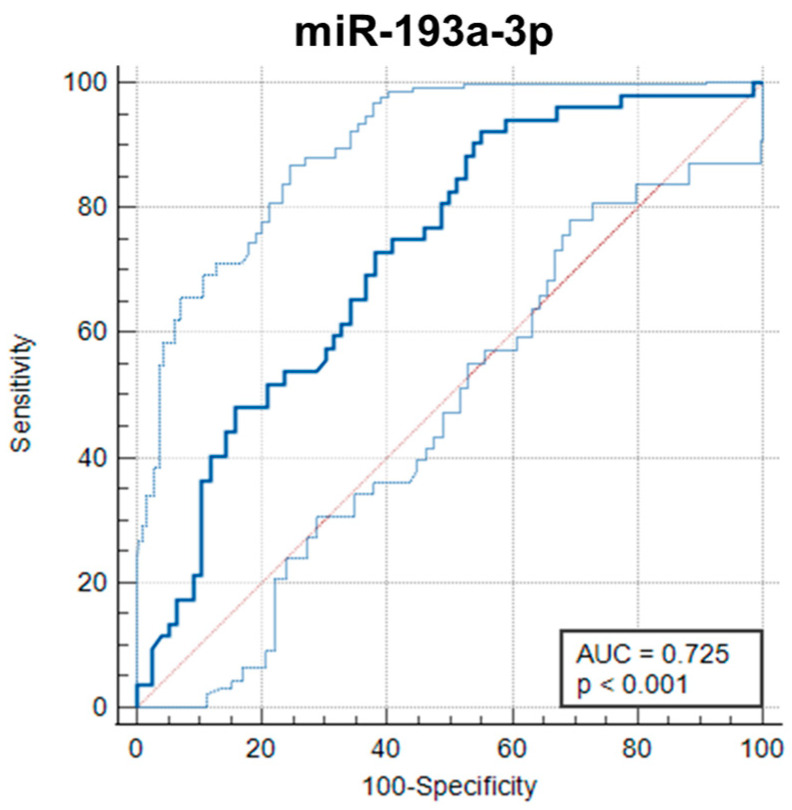
The ROC curve for expression levels of miR-193a-3p in FFPE tissue samples. MiR-193a-3p showed biomarker potential for discriminating CRC and pre-CRC patients with an AUC of 0.725 (95% CI = 0.640–0.801, *p* < 0.0001). The dark blue curve represents the receiver-operating characteristic (ROC) curve. The light blue curve represents a 95% ROC confidence interval. The red line represents a diagonal.

Furthermore, we performed ROC curve analysis to evaluate the potential diagnostic value of miRNAs in tissue samples. MiR-193a-3p showed biomarker potential for discriminating CRC and pre-CRC patients with an AUC of 0.725 (95% CI = 0.640–0.801, *p* < 0.0001) (Figure 5), while miR-19a-3p, miR-92a-3p, and miR-210-3p had poor performance with AUCs of 0.542 (95% CI = 0.452–0.630, *p* = 0.434), 0.561 (95% CI = 0.471–0.649, *p* = 0.2311), and 0.547 (95% CI = 0.457–0.6350, *p* = 0.3794), respectively.

### 2.4. Logistic Regression

A pairwise comparison of ROC curves was made because we wanted to assess if the combination of tumor marker concentrations and expression levels of chosen miRNAs in tissues and exosomes could be better for the discrimination of pre-CRC and CRC patients than only using concentrations of tumor markers already in clinical practice (Table S1). The best-performing model was shown to be a combination of CEA concentration and the expression level of miR-193a-3p in tissues. The AUC was 0.823 (95% CI = 0.741–0.888, *p* < 0.0001). As a result, those two markers could correctly classify CRC and pre-CRC in 71.3% of cases (Table S2).

## 3. Discussion

According to data from the Croatian Institute of Public Health, CRC is Croatia’s the most common malignant disease with increasing incidence and mortality rates. It is the third most common cancer in the world, after breast cancer and lung cancer, as well as the fourth most lethal [2,26,27].

CRC is more common in older adults, which we have confirmed in this research. CRC is preceded by colorectal adenoma, a still benign condition that we used as a control condition. Colonoscopy is the gold standard in diagnosing CRC, during which a classic biopsy is performed. It is an invasive and painful procedure with a high cost and risk of complications that require bowel preparation, which is unpleasant for the patients [3,28,29].

CEA and CA 19-9 are tumor markers used in clinical practice for prognosis and prediction, follow-up after surgery, and monitoring of CRC. These markers are characterized by insufficient sensitivity and specificity because they are elevated in other cancers and in some benign conditions [30]. We proved that they are valuable for discriminating against CRC from pre-CRC patients. CEA had higher specificity and sensitivity, as expected. Their serum concentrations can be used with other diagnostic tools such as a pathohistological analysis that patients undergo during recruitment [31].

The pathological stage of CRC determines the survival of patients, and it is estimated that if the cancer is detected in the earlier stages, the patient’s survival rate is 90% [32]. The pathohistological parameters indicate that the concentration of tumor markers should be higher in the advanced stages of the CRC, but we found no statistical association between pathohistological parameters and CEA or CA 19-9.

Unfortunately, there are no early symptoms of CRC and, as we can see, the existing methods and markers have numerous flaws and limitations. Consequently, the need for the discovery of new markers that would help in the early diagnosis, distinguishing pre-CRC from CRC, as well as in timely treatment clearly arises.

MicroRNAs appear to be promising markers of various cancers, including CRC, so it is important to find CRC-related miRNAs. In this research, we compared expression levels of miR-19a-3p, miR-92a-3p, miR-193a-3p, and miR-210-3p between tissue samples of CRC and pre-CRC patients. A statistically significant difference was found only for miR-193a-3p.

The dysregulation of miR-193a-3p, a microRNA we focused on in our research, has been observed in a diverse range of cancers, including oral cancer, osteosarcoma, and pleural mesothelioma. This wide spectrum of cancers where miR-193a-3p is implicated underscores its significant role in tumorigenesis, making our findings even more compelling [33,34,35]. As reported in the literature, miR-193a-3p has a role in cell growth inhibition, migration, and invasion and induces cell apoptosis by targeting PAK3 in CRC [36].

We showed that miR-193a-3p had higher expression levels in CRC tissues. Data from research by Zhao et al. indicate that miR-193a-3p was overexpressed in CRC tissue samples and its high expression predicted poor overall survival [37].

In contrast, Mamoori et al. found that the miR-193a-3p expression was predominantly downregulated in the CRC tissues, but they also showed overexpression of miR-193a in colon cancer cells after stable transfection [38]. On the other hand, Lin et al. manifested that miR-193a-3p expression was decreased in CRC cell lines and that its upregulation inhibited tumor development and progression in vitro by regulating cell growth, migration, and angiogenesis partly through targeting the PLAU pathway [22]. Lin et al. proved that miR-193a-3p was differentially expressed between E-cadherin-negative and E-cadherin-positive CRC tissues [39].

During our research, we performed ROC curve analysis to evaluate the potential diagnostic value of expression levels of four chosen miRNAs in tissue samples. The results showed that only miR-193a-3p could be a valuable biomarker for discriminating against CRC and pre-CRC patients. Motoyama et al. showed that miR-92 had a higher expression level in CRC than in non-CRC tissues [40]. Still, we did not find a statistically significant difference in expression levels between CRC and pre-CRC patients neither for miR-92a-3p, miR-19a-3p, or miR-210-3p. In addition, Romanowicz et al. did not find a statistically significant difference in the miR-210 expression among CRC patients or in the control group either [41]. On the other hand, Ng et al. demonstrated that miR-92 plasma levels were valuable biomarkers for differentiating patients with CRC from healthy controls [42]. Our study, while not yielding the desired results, is a valuable contribution to the field, as it provides evidence that miR-92a-3p, miR-19a-3p, and miR-210-3p are not effective biomarkers for distinguishing CRC and pre-CRC patients.

As mentioned before, the diagnosis of CRC is based on painful and invasive methods. For this purpose, it is necessary to develop less invasive methods that are more compliant for the patients and reproducible. Liquid biopsy seems like a promising tool for improving the diagnosis, prognostication, and monitoring of CRC [43].

In our study, we took a unique approach to demonstrate the potential benefit of liquid biopsy. We compared the expression levels of miRNAs not only between CRC and pre-CRC tissue samples but also between tissues and exosomes isolated from the plasma of CRC patients. It is noteworthy that our findings align with those of Dohmen et al., who observed enrichment in the total and single miRNAs in the exosomal compartment compared to free circulating miRNAs in serum [44]. Accordingly, exosomes were the most promising source for our miRNA analyses.

We found statistically significant differences comparing the tissues and exosomes expression levels of miR-19a-3p, miR-92a-3p, miR-193a-3p, and miR-210-3p.

Yong et al. observed that dysregulations in circulating blood miR-193a-3p, miR-23a, and miR-338-5p reflect those in CRC tissues and could be used as potential blood biomarkers for early detection of CRC [45]. Pesta et al. discovered that nine miRNAs, including miR-92a-3p and miR-210-3p, displayed a higher sensitivity and lower specificity than CEA and CA 19-9 as biomarkers of CRC recurrence [46].

We must also note that we did not find statistical significance when comparing miRNA expression levels in tissues or exosomes and pathohistological parameters—similar to the study of Conev et al. [47]. Likewise, Mamoori and colleagues also did not find a significant correlation between miR-193a-3p expression and pathological parameters [38].

Other studies have shown that the upregulation of miR-210 and miR-92a and downregulation of miR-193a-3p were associated with the advanced pTNM stage of CRC, tumor growth, and the presence of lymph node metastasis as well [39,48,49]. Other miRNAs, such as miR-21 and miR-223, were also found to be associated with pathohistological parameters [32,50].

Our main goal was to assess whether the combination of tumor marker concentrations and expression levels of chosen miRNAs in tissues and exosomes is better than only using the CRC biochemical gold standards CEA and CA 19-9. For this reason, we made pairwise comparisons of ROC curves and, in the final logistic regression analysis model, we kept only the CEA concentration and the miR-193a-3p expression level in tissues. As a result, we obtained that by using these two markers, we can distinguish CRC and pre-CRC in 71.3% of cases. These findings open new avenues for understanding cancer progression.

Although our results are promising, this study has several limitations. The greater significance of the final outcomes would be achieved if the analyses were performed on more patients. Also, it would be desirable to perform miRNA isolation from pre-CRC exosomes to make ROC curve analyses for those samples. In this way, the importance of liquid biopsy would be better evaluated. Therefore, we will consider such approaches when planning our future experiments to elucidate these devastating gut diseases for which the unmet medical need still exists.

Our findings, which did not show a statistically significant difference when comparing standard tumor marker (CEA and CA 19-9) concentrations with pathohistological parameters, highlight the pressing need for more specific biomarkers. These could potentially serve as more accurate and reliable parameters in the diagnosis and follow-up of patients affected by CRC and pre-CRC, a crucial area for future research.

However, our work presents a novel finding: miR-193a-3p from tissue samples could potentially serve as a significant biomarker, distinguishing between CRC and pre-CRC conditions. This discovery opens new avenues for research and underscores the importance of our study’s findings.

## 4. Materials and Methods

The research was carried out at the Department of Medical Biochemistry and Hematology, University of Zagreb Faculty of Pharmacy and Biochemistry; Department of Internal Medicine—Division of Gastroenterology and Hepatology; and Department of Clinical Chemistry and Department of Pathology and Cytology University Hospital Centre Sestre Milosrdnice Zagreb, Croatia.

The study was conducted in accordance with the Declaration of Helsinki and the protocol was approved by the Ethics Committee for Experimentation of the University of Zagreb Faculty of Pharmacy and Biochemistry (approval no. 251-62-03-19-29) and the Ethics Committee of the University Hospital Center Sestre Milosrdnice, Zagreb, Croatia (approval no. EP-19243/17-6).

### 4.1. Study Population

Clinical subjects were recruited among the patients undergoing colonoscopy due to suspicion of CRC. The participants signed the informed consent in compliance with the approved study protocol. Upon completing diagnostic procedures, participants were subjected to a colonoscopy. Based on the colonoscopy findings and subsequent histopathological analysis of the specimen excised (by resection or a later surgical procedure in case of CRC), they were divided into two groups: (1) subjects with CRC (CRC subjects) and (2) subjects with adenoma (pre-CRC subjects). According to the study strength estimation, the study included 52 CRC subjects and 76 pre-CRC subjects.

Blood samples of 52 CRC patients were collected in K3EDTA tubes, CellSave tubes, and tubes without anticoagulants. These were further processed to obtain plasma, circulating tumor cells (CTCs), and serum. We used plasma samples to isolate exosomes (separating and analyzing proteins and miRNAs). In addition, for further investigation and comparison, CEA and CA 19-9 concentrations were determined in 49, i.e., 48 CRC and 67 pre-CRC subjects’ serum samples on the e401 analyzer (Roche Diagnostics, Rotkreuz, Switzerland).

Serum/plasma/blood and tissue are often compared samples in medical research to understand how diseases and treatments affect the body on a cellular level. For this reason, our study also included tissue sampling. Tissue samples were formalin-fixed and paraffin-embedded (FFPE) and DNA, RNA, and miRNA were isolated from them.

### 4.2. Isolation of Tissue and Circulating miRNAs

MiRNA from 76 pre-CRC and 52 CRC FFPE tissue samples was isolated according to the manufacturer’s guidelines, utilizing the miRNeasy FFPE Kit (Qiagen, Hilden, Germany). For exosome isolation, plasma was separated from whole blood samples by differential centrifugation in a centrifuge LISA (AFI, Château-Gontier, France) at 4 °C. Exosomes were isolated from the cell-free supernatant with low platelet content using the miRCURY Exosome Serum/Plasma Kit (Qiagen, Hilden, Germany) according to the manufacturer’s protocol. The MiRNeasy Serum/Plasma Advanced Kit (Qiagen, Hilden, Germany) was used for exosomal miRNA isolation, which was performed on the same day as the exosome isolation, following the manufacturer’s guidelines. The concentrations of the isolated miRNA were determined using the DS-11 spectrophotometer (DeNovix, Wilmington, DE, USA).

### 4.3. qPCR (RT-qPCR)

Four miRNAs, miR-19a-3p, miR-92a-3p, miR-193a-3p, and miR-210-3p, were selected to characterize CRC and distinguish it, namely, from pre-CRC. MiR-103a-3p was used as a reference and UniSp6 was used as an internal control.

Reverse transcription of miRNAs into cDNA was performed with the miRCURY RT LNA Kit (Qiagen, Hilden, Germany). The expression levels of miRNAs were analyzed using the 7500 Real-Time PCR System (Applied Biosystems, Foster City, CA, USA) with the miRCURY LNA SYBR GREEN PCR Kit (Qiagen, Hilden, Germany) and miRCURY LNA miRNA PCR Assays miR-19a-3p (YP00205862), miR-92a-3p (YP00204258), miR-193a-3p (YP00204591), miR-210-3p (YP00204333), miR-103a-3p (YP00204063), and UniSp6 (YP00203954) (Qiagen, Hilden, Germany).

### 4.4. Pathohistological Analysis

Pathohistological analysis was carried out using current national guidelines and recent publications [51,52,53]. The pathohistological findings include data on the tumor size, degree of malignancy, presence, and depth of penetration through the wall, the number of examined and tumor-affected regional lymph nodes, possible penetration of the tumor through the capsule, and information on the existence of invasion of lymph and blood vessels, i.e., perineural invasions, presence of the tumor cells in pericolic fat tissue outside lymph nodes (tumor deposits), the condition of proximal and/or distal cutting edge, circumferential edge, mesenteric edge, stapler ring edges, the status of mesorectal fascia (complete excision, almost complete excision or incomplete excision), and the existence of microsatellite instabilities (MSI or MSS). Of all the listed parameters, we only took MSI, pathological Tumor-Node-Metastasis (pTMN) stage, gradus, tumor subtype, and its size in centimeters in the statistical analysis.

### 4.5. Statistical Analysis

The expression levels of miRNAs were estimated using the 2^−ΔCt^ values and log_2_fold change (log_2_FC) was used for the graphical representations. All numeric data were tested for normality by the Kolmogorov–Smirnov test and failed it so non-parametric statistical tests were performed. Data were presented as the median with an interquartile range; only the age was presented as the median with a minimum and maximum. To distinguish the expression levels of miRNAs and concentrations of CEA and CA 19-9 between CRC and pre-CRC patients, i.e., their tissue samples, the Mann–Whitney test was used. To compare expression levels between tissue and exosome samples from CRC patients, the Wilcoxon matched-pairs test was used. The diagnostic utility of CEA and CA 19-9 concentrations was evaluated by the receiver-operating characteristic (ROC) curve and area under the ROC curve (AUC) analysis. The potential diagnostic value of chosen miRNAs isolated from tissue samples in CRC was also evaluated by the ROC curve and AUC analysis. A *p* value < 0.05 in ROC curve analysis signifies that the ROC curve is significantly different from 0.5 (diagonal) and therefore that there is evidence that a test could distinguish between two groups.

We wanted to assess whether combining tumor marker concentrations and expression levels of chosen miRNAs in tissues and exosomes is better than using only concentrations of clinical practice-used tumor markers. We made pairwise comparisons of ROC curves using the DeLong test. In the final logistic regression analysis model, we kept only data that showed statistically significant results. Pathohistological parameters were compared with the expressions of chosen miRNAs using the Kruskal–Wallis test.

Statistical analysis was performed using MedCalc Statistical Software, v22.020 (MedCalc Software Ltd., Ostend, Belgium). A *p*-value < 0.05 was considered statistically significant. The Bonferroni method, which takes multiple tests into account and reduces the risk of false-positive results, was used as the correction method.

## Figures and Tables

**Table 1:** The age and concentrations of tumor markers. Age was shown as the median with a minimum and maximum. CA 19-9 concentrations were measured on * 67 pre-CRC and ** 48 CRC serum samples. CEA concentrations were measured in * 67 pre-CRC and *** 49 CRC serum samples and shown as the median and interquartile range.

Parameter	pre-CRC Controls (n = 76)	CRC Patients (n = 52)	*p*-Value
Age	67 (32–84)	72 (37–90)	0.0019
CA 19-9 (kIU/L)	5.81 (2.59–11.72) *	9.03 (6.70–18.95) **	<0.0001
CEA (μg/L)	1.73 (1.23–2.67) *	3.50 (2.41–7.55) ***	<0.0001

**Table 2:** Pathohistological data for CRC patients.

Pathohistological Parameter	Pathohistological Subcategory	% of CRC Patients
MSI	MSS	82.5
	MSI-H	17.5
pTMN stage	1	30
	2	32
	3	32
	4	6
gradus	Low	95.8
	high	4.2
subtype	NOS	98
	mucinous	2
tumor size in cm	≤2	12
	>2 ≤ 5	68
	>5 ≤ 10	20

MSI—Microsatellite Instability; MSS—Microsatellite Stable; MSI-H—Microsatellite Instable-High; pTMN—pathological Tumor-Node-Metastasis; NOS—No Other Specified.

## Data Availability

Data are available on request from the authors.

## Supplementary Materials

The supporting information can be downloaded at https://www.mdpi.com/article/10.3390/ijms25158156/s1.
