# Supplementary material for: MicroRNA-193a-3p as a Valuable Biomarker for Discriminating between Colorectal Cancer and Colorectal Adenoma Patients
Source: Int J Mol Sci. 2024 Jul 26;25(15):8156. doi: 10.3390/ijms25158156 (PMC11311302; doi:10.3390/ijms25158156)
Supplement: Supplementary file 1 [file ijms-25-08156-s001.zip › ijms-3097664-supplementary.pdf]

Supplemental File

Table S1: Pairwise comparison of ROC curves

Variable	AUC	SE	95% CI	P (vs. CEA)
CA-19.9	0.668	0.0507	0.574 to 0.753	0.0245
CEA	0.796	0.0421	0.711 to 0.866	
AGE	0.623	0.0522	0.533 to 0.707	0.0351
miR-193a	0.725	0.0448	0.640 to 0.801	0.8325

Table S2: Logistic regression final model

Coefficients and Standard Errors

Variable	Coefficient	Std. Error	Wald	P
CEA	0.47996	0.13346	12.9331	0.0003
miR-193a_	33.84185	15.71223	4.6391	0.0313
Constant	-2.27118	0.47453	22.9072	<0.0001

Percent of cases correctly classified	71.30%

Area under the ROC curve (AUC)	0.823
Standard Error	0.0391
95% Confidence interval	0.741 to 0.888
